# Identification of Pre-Renal and Intrinsic Acute Kidney Injury by Anamnestic and Biochemical Criteria: Distinct Association with Urinary Injury Biomarkers

**DOI:** 10.3390/ijms24031826

**Published:** 2023-01-17

**Authors:** Sandra M. Sancho-Martínez, Alfredo G. Casanova, Annette G. Düwel, Karen Rivero-García, Tamara García-Garrido, Ana I. Morales, Carlos Martínez-Salgado, Francisco J. López-Hernández, Pilar Fraile

**Affiliations:** 1 Institute of Biomedical Research of Salamanca (IBSAL), 37007 Salamanca, Spain; 2 Departamento de Fisiología y Farmacología, Universidad de Salamanca, 37007 Salamanca, Spain; 3 Group of Translational Research on Renal and Cardiovascular Diseases (TRECARD), 37007 Salamanca, Spain; 4 National Network for Kidney Research RICORS2040 RD21/0005/0004, Instituto de Salud Carlos III, 28029 Madrid, Spain; 5 Instituto de Estudios de Ciencias de la Salud de Castilla y León (IECSCYL), 42002 Soria, Spain; 6 Servicio de Nefrología, Complejo Asistencial Universitario de Salamanca, 37007 Salamanca, Spain; 7 Group of Biomedical Research on Critical Care (BioCritic), 47003 Valladolid, Spain

**Keywords:** acute kidney injury, pre-renal, intrinsic, injury biomarkers, anamnesis, etiopathology

## Abstract

Acute kidney injury (AKI) is a syndrome of sudden renal excretory dysfunction with severe health consequences. AKI etiology influences prognosis, with pre-renal showing a more favorable evolution than intrinsic AKI. Because the international diagnostic criteria (i.e., based on plasma creatinine) provide no etiological distinction, anamnestic and additional biochemical criteria complement AKI diagnosis. Traditional, etiology-defining biochemical parameters, including the fractional excretion of sodium, the urinary-to-plasma creatinine ratio and the renal failure index are individually limited by confounding factors such as diuretics. To minimize distortion, we generated a composite biochemical criterion based on the congruency of at least two of the three biochemical ratios. Patients showing at least two ratios indicative of intrinsic AKI were classified within this category, and those with at least two pre-renal ratios were considered as pre-renal AKI patients. In this study, we demonstrate that the identification of intrinsic AKI by a collection of urinary injury biomarkers reflective of tubular damage, including NGAL and KIM-1, more closely and robustly coincide with the biochemical than with the anamnestic classification. Because there is no gold standard method for the etiological classification of AKI, the mutual reinforcement provided by the biochemical criterion and urinary biomarkers supports an etiological diagnosis based on objective diagnostic parameters.

## 1. Introduction

Acute kidney injury (AKI) is a syndrome of sudden renal excretory dysfunction with serious sanitary and economic consequences [1,2], consuming 1% of the total health budget [3] and 5% of hospital expenditures [4,5]. The immediate impact of AKI is very variable and particularly pernicious in the intensive care setting, where incidence and mortality may reach 30–50% [1,6] and 40–80% [6,7,8,9,10], respectively. Defective recovery from AKI is also associated with long-term morbidity and mortality [11,12], including permanent dependence on dialysis (in 12.5% of the cases) [13] and progression to chronic kidney disease (CKD) [14,15]. Furthermore, apparently fully recovered patients bear a lower, but significantly increased, risk of future health complications [11,12,16].

The prognosis of AKI patients is determined by previous comorbidities, including chronic kidney disease (CKD), as well as the severity [17,18] and etiopathology of the AKI episode [1,19,20]. Regarding its etiopathology, AKI is most commonly classified into pre-renal, renal (or intrinsic) and post-renal (obstructive) sub-types [21,22,23,24], each of which requires distinct handling and prognosis [1,19,20]. Pre-renal AKI is a syndrome of renal hemodynamic deficit in which kidney structures are preserved whilst intrinsic forms feature parenchymal damage, most commonly of the tubular structures. Consequently, pre-renal AKI is associated with a more favorable clinical outcome than intrinsic AKI [21,25,26,27,28,29].

Distinction of AKI types may be, in practice, a complicated task. The gold standard diagnostic biomarker (i.e., plasma creatinine concentration, Cr_p_) provides no etiological information, as it increases in all forms of AKI [1,18]. Indeed, undamaged renal parenchyma may be found with all levels of Cr_p_, and Cr_p_ may be found to be normal through a range of parenchymal damage [30,31]. Etiological identification is frequently obscured by multi-causality. When different potential causes of AKI concomitantly occur, multiple pathological combinations and damage patterns may underly them. In an undetermined number of pre-renal cases, damage may progress to renal damage through a complex continuum that further complicates diagnosis [32,33].

Traditionally, etiopathological stratification has been approached retrospectively, with variable and undetermined success, based on the anamnestic evaluation of the duration of the episode, the response to fluid therapy [23,24,28,29,33] and, occasionally, on microscopic analysis of the urinary sediment [34,35,36]. In the absence of more objective criteria, anamnesis has proved, with limitations, to be a valuable tool to determine AKI etiology and, based on it, to define the best therapeutic approach. Biochemical parameters of tubular performance, such as the fractional excretion of sodium (FENa) and urea (FEU) [37], as well as other ratios involving plasma and urinary urea and creatinine [38], have also been used. These parameters may potentially provide more objective criteria, but their utility has been disputed [32], as confounding factors (e.g., diuretics, contrast media, volemic and hydration status, CKD, bicarbonaturia, glycosuria, Addison disease and renal damage secondary to myoglobin/hemoglobin) may alter their significance. More recently, a few pre-clinical (and some clinical) studies have shown that the urinary levels of calprotectin and neutrophil gelatinase-associated lipocalin (NGAL) [39,40,41], activin A [42], klotho and S100A8/A9 [43] might distinguish pre-renal from renal AKI. In general, “injury biomarkers” (e.g., NGAL, kidney injury molecule 1 (KIM-1), tissue inhibitor of metalloproteinase-2 (TIMP-2) and insulin-like growth factor binding protein 7 (IGFBP7)) are proposed to be shed by damaged renal structures and, thus, to discriminate AKI forms with variable success [1,44,45]. In fact, at least in animal models displaying pure syndromes, injury biomarkers should be absent in pre-renal and present in intrinsic forms of AKI [46].

Etiopathological diagnosis of AKI is still limited by the absence of verification procedures. Renal biopsy is not a routine, but an occasional practice, and it would provide only a limited discrimination capacity, as some sublethal alterations may not be evident in histological specimens. Accordingly, the absence of a non-invasive gold standard to define pre-renal AKI or to distinguish between AKI types makes it difficult (or impossible) to compare efficacy between diagnostic methods and to reliably accomplish differential diagnosis. On these grounds, with a mutual-reinforcement approach, the robustness of the anamnestic and biochemical criteria for etiopathological diagnosis was examined through their association with urinary injury biomarkers.

## 2. Results

### 2.1. Patient Description and Etiological Classification

**Figure 1:**
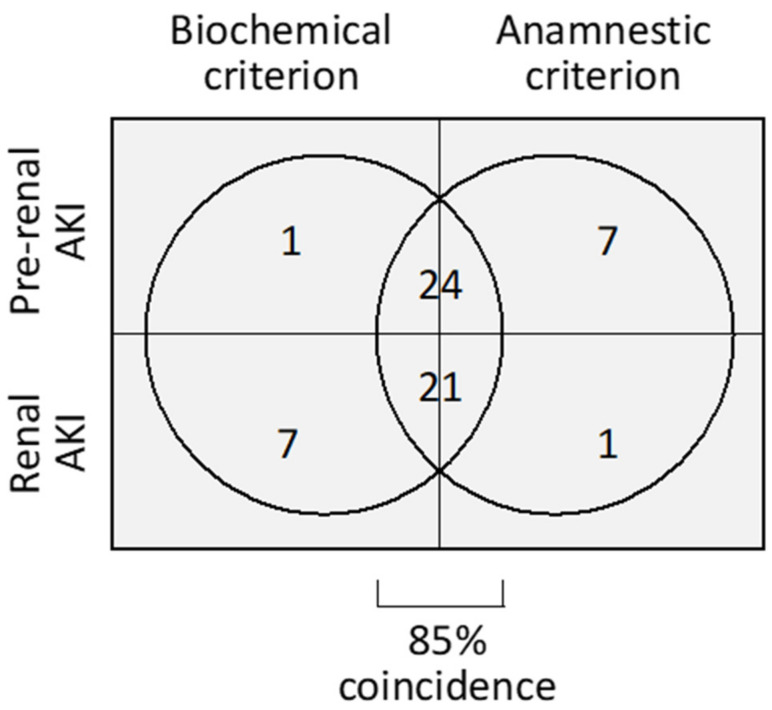
Distribution of pre-renal and renal AKI patients according to the biochemical and anamnestic criteria.

The characteristics of the patients included in this study per type of AKI (i.e., pre-renal or renal) according to anamnestic and biochemical criteria (Figure 1) are shown in Table 1. No significant differences in age, sex, comorbidity or drug treatment existed between pre-renal and renal AKI patients when classified by either of the two criteria.

### 2.2. Evaluation of Urinary Biomarkers

**Figure 2:**
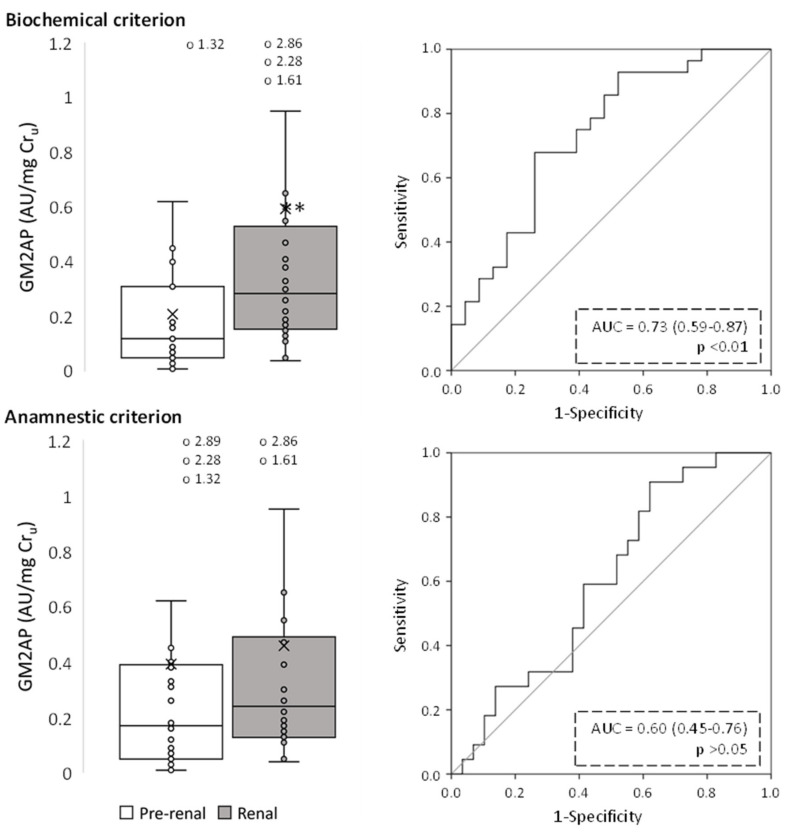
Urinary GM2AP levels of pre-renal and renal AKI patients following classification by biochemical and anamnestic criteria, shown as box plots (**left** panels) representing median values in arbitrary units (AU) of urinary GM2AP per mg urinary creatinine (Cr_u_), and ROC curves (**right** panels). AUC: area under the ROC curve showing the pre-renal/renal classification efficacy of urinary GM2AP. The × in box plots represents the median value. **, *p* < 0.01.

**Figure 3:**
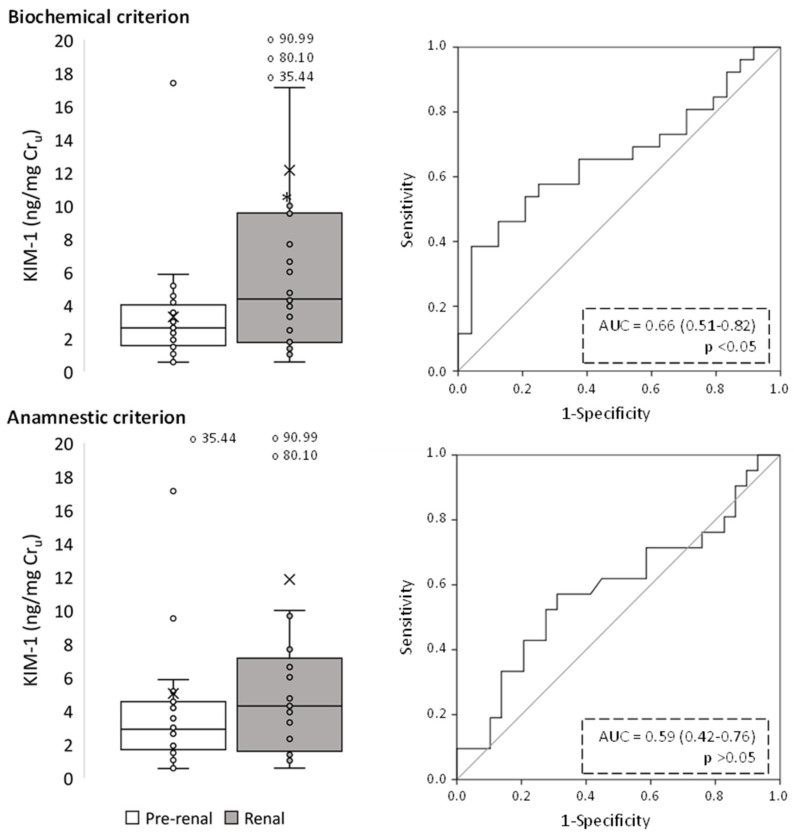
Urinary KIM-1 levels of pre-renal and renal AKI patients following classification by biochemical and anamnestic criteria, shown as box plots (**left** panels) representing median values in ng of urinary KIM-1 per mg urinary creatinine (Cr_u_), and ROC curves (**right** panels). AUC: area under the ROC curve showing the pre-renal/renal classification efficacy of urinary KIM-1. The × in box plots represents the median value. *, *p* < 0.05.

**Figure 4:**
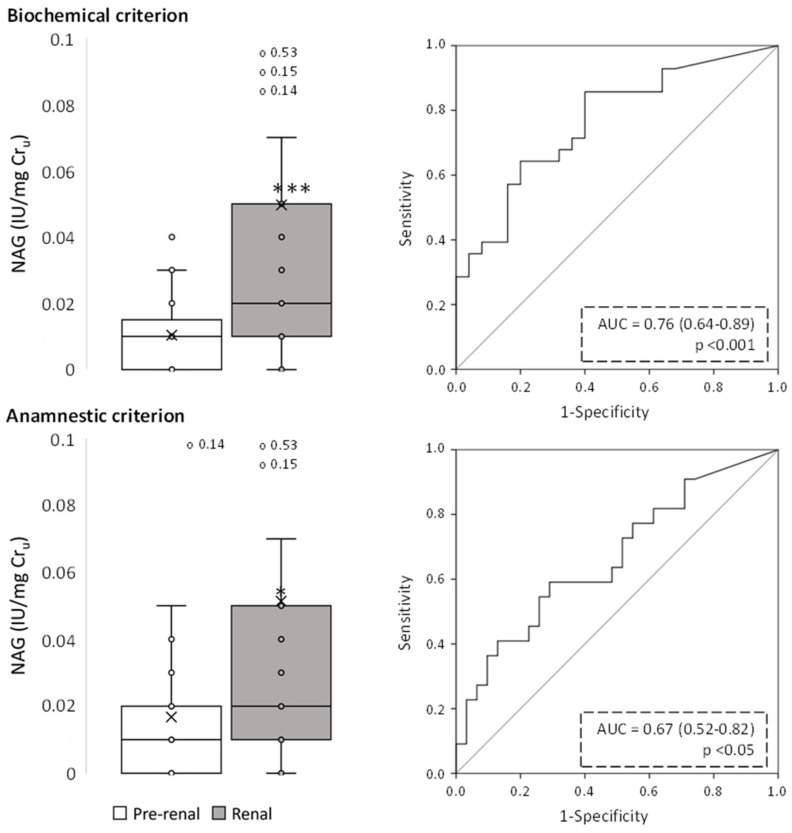
Urinary NAG levels of pre-renal and renal AKI patients following classification by biochemical and anamnestic criteria, shown as box plots (**left** panels) representing median values in international units (IU) of urinary NAG per mg urinary creatinine (Cr_u_), and ROC curves (**right** panels). AUC: area under the ROC curve showing the pre-renal/renal classification efficacy of urinary NAG. The × in box plots represents the median value. *, *p* < 0.05; ***, *p* < 0.001.

**Figure 5:**
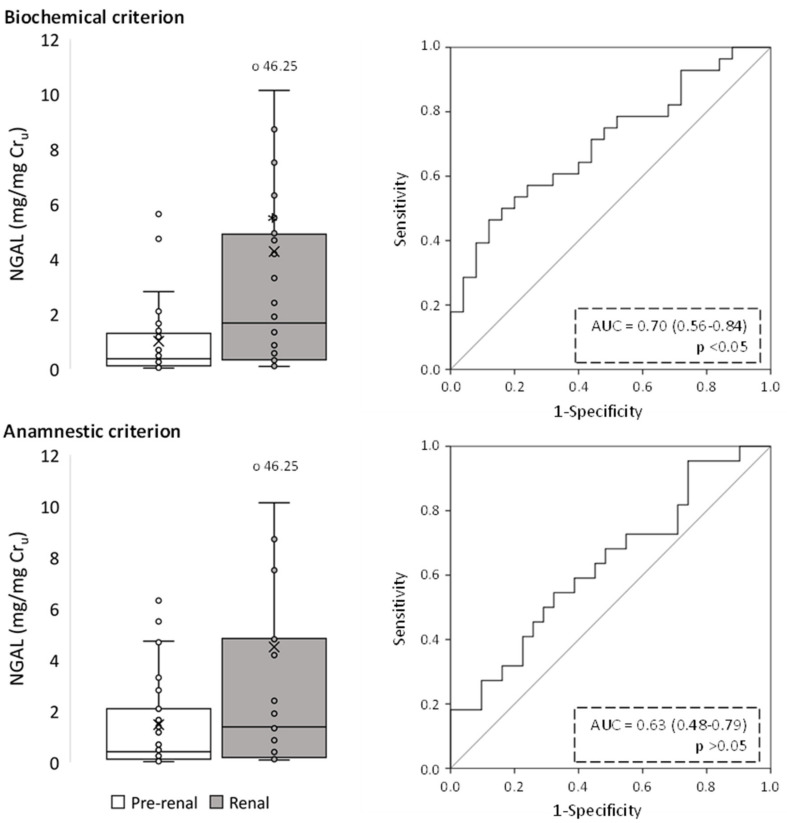
Urinary NGAL levels of pre-renal and renal AKI patients following classification by biochemical and anamnestic criteria, shown as box plots (**left** panels) representing median values in mg of urinary NGAL per mg urinary creatinine (Cr_u_), and ROC curves (**right** panels). AUC: area under the ROC curve showing the pre-renal/renal classification efficacy of urinary NGAL. The × in box plots represents the median value. *, *p* < 0.05.

**Figure 6:**
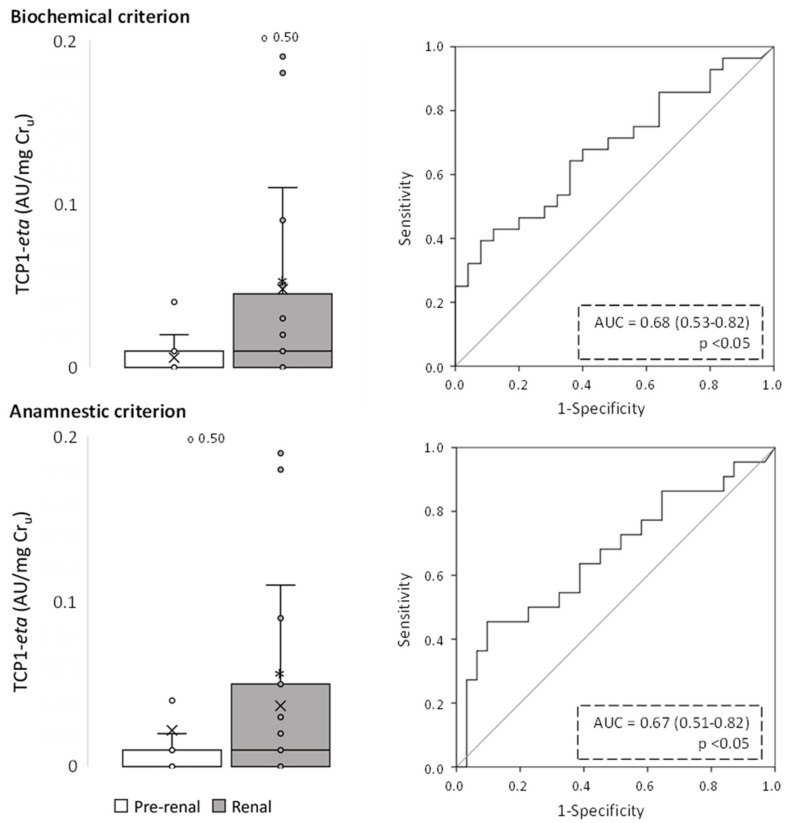
Urinary TCP1-eta levels of pre-renal and renal AKI patients following classification by biochemical and anamnestic criteria, shown as box plots (**left** panels) representing median values in arbitrary units (AU) of urinary TCP1-eta per mg urinary creatinine (Cr_u_), and ROC curves (**right** panels). AUC: area under the ROC curve showing the pre-renal/renal classification efficacy of urinary TCP1-eta. The × in box plots represents the median value. *, *p* < 0.05.

**Figure 7:**
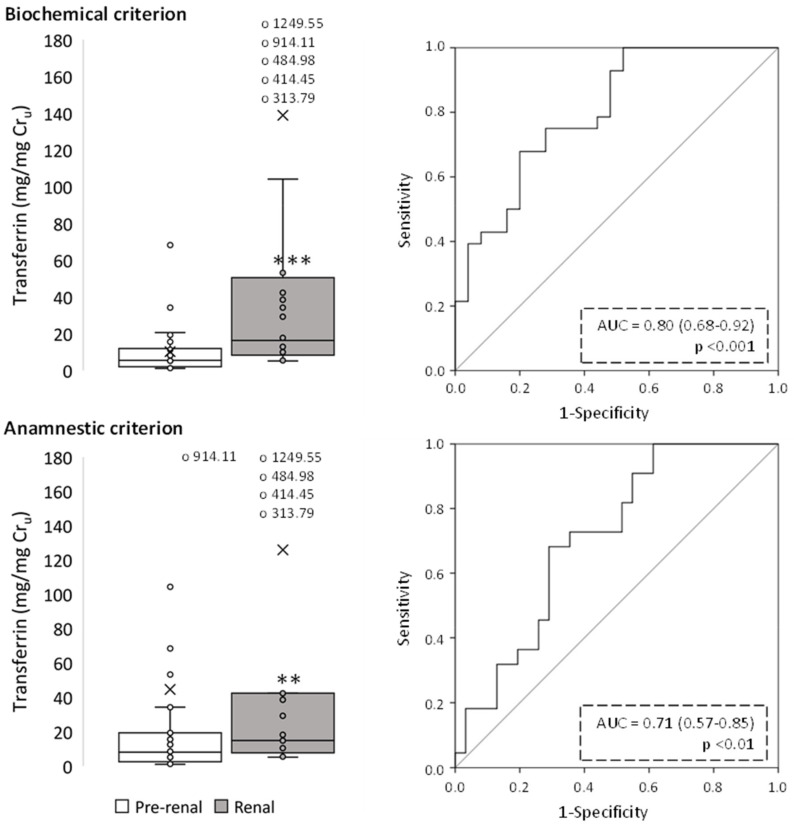
Urinary transferrin levels of pre-renal and renal AKI patients following classification by biochemical and anamnestic criteria, shown as box plots (**left** panels) representing median values in mg of urinary transferrin per mg urinary creatinine (Cr_u_), and ROC curves (**right** panels). AUC: area under the ROC curve showing the pre-renal/renal classification efficacy of urinary transferrin. The × in box plots represents the median value. **, *p* < 0.01; ***, *p* < 0.001.

**Figure 8:**
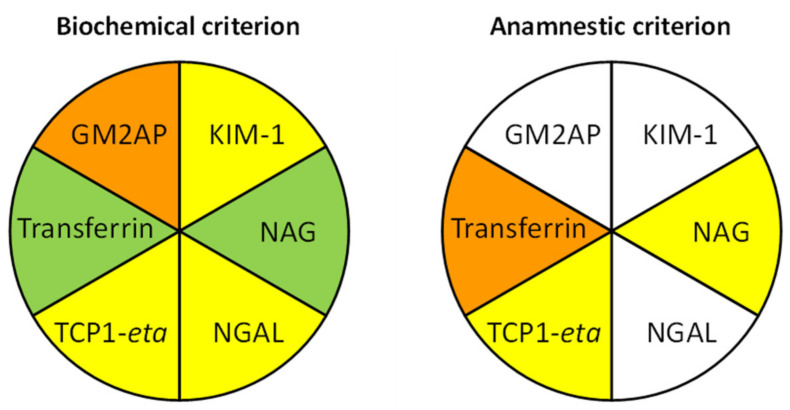
Summary of the etiological diagnostic capacity of urinary injury biomarkers (according to the area under the ROC curve) following pre-renal/renal classification by biochemical and anamnestic criteria. Color key: yellow, *p* < 0.05; orange, *p* < 0.01; green, *p* < 0.001; white, *p* > 0.05.

Figure 2, Figure 3, Figure 4, Figure 5, Figure 6 and Figure 7 show the excretion of GM2AP, KIM-1, NAG, NGAL, TCP1-eta and transferrin, respectively, as well as the analysis of their predictive capacity based on ROC curves in patients with pre-renal and renal AKI, according to both classification criteria. A summary of their diagnostic abilities is presented in Figure 8. When biochemical criteria were applied, a significantly higher excretion of NAG, transferrin (*p* < 0.001), GM2AP (*p* < 0.01), KIM-1, NGAL and TCP1-eta (*p* < 0.05) was observed in patients with renal-type AKI. However, after applying the criteria based on anamnesis, the only biomarkers significantly elevated in patients with renal AKI were transferrin (*p* < 0.01), NAG and TCP1-eta (*p* < 0.05). For both criteria, the biomarker that presented a better predictive capacity according to its ROC curve was transferrin, but the area under the curve (AUC) was higher for the biochemical criterion (0.80, *p* < 0.001) than for the anamnestic (0.71, *p* < 0.01).

The binary logistic regression analysis with which we intended to obtain the best combination of biomarkers that would allow for discrimination between patients with renal AKI from those with pre-renal AKI (Table 2) generated a significant model, after applying the biochemical classification criteria, for transferrin (specificity: 81.8%; sensitivity: 61.5%; percentage of success: 70.8%). The model’s sensitivity and percentage of success improved when including the biomarker NAG (specificity: 77.3%; sensitivity: 76.9%; percentage of success: 77.1%). In contrast, no significant logistic regression model was obtained when the anamnestic classification criterion was applied.

### 2.3. Evaluation of the Influence of Diuretic Treatment on Patient Classification Mismatch

The analysis of contingency tables ruled out an influence of diuretics on the differences observed in the classification of some patients by anamnestic and biochemical criteria (Table 3).

## 3. Discussion

The search for parameters performing objectively for the etiopathological diagnosis of AKI is conceptually flawed, as candidates are almost invariably validated against anamnesis as the standard. Parameters providing results deviating from the anamnestic classification are consequently and inevitably deemed as less effective, even if they might actually perform more accurately. Renal biopsies are rarely obtained due to legal and medical restrictions, and these do not bestow a standard, as parenchymal alterations not affecting the gross renal structure may pass unnoticed to pathological examination. The absence of a recognized standard thus makes it impossible to ascertain the absolute utility of new criteria.

To overcome this limitation, we studied the congruency of three criteria of distinction between pre-renal and intrinsic AKI (i.e., anamnestic, biochemical and based on injury biomarkers) in internal, relative terms. In our study cohort, the anamnestic and biochemical criteria largely (i.e., in 85% of the cases), but not completely, coincided. The discrepancy (i.e., the other 15%) could not be explained by diuretics confounding the meaning of biochemical ratios. Triage provided by the level of six urinary renal injury biomarkers (i.e., NAG, NGAL, KIM-1, GM2AP, TCP1-*eta* and transferrin) more closely and more robustly associated with the biochemical than with the anamnestic classification. We contend that one key aspect of our approach is the multifactorial nature of the biochemical criterion. While each biochemical ratio may be individually affected by a determined external confounder, it is more unlikely that two out of the three ratios became distorted by the same factor. Therefore, patients should be better classified according to a flexible criterion buffering potential discrepancies (i.e., two out of three ratios) than by rigid criteria such as those based on a single ratio or on the coincidence of the three ratios. Additional biochemical ratios (such as the fractional excretion of urea) and biomarkers to those used in this study should be added to new studies. In perspective, the ultimate goal should be to associate molecular patterns (i.e., biochemical and biomarker fingerprints) to specific clinical features and outcomes.

However, molecular patterns must also be interpreted with caution, as biomarkers and biochemical ratios may conceal diffuse ambiguity. The distinction between pre-renal and renal AKI is based on tubular performance. Tubular dysfunction causing biochemical ratios consistent with intrinsic AKI may result from tubular necrosis or from sublethal functional alterations [47]. The short- and long-term prognosis, evolution, and outcome are expected to differ substantially between intrinsic AKI subtypes involving extensive structural damage and those limited to tubular dysfunction which retain structural integrity. In addition, both subtypes may be primary causes of intrinsic AKI, or secondary consequences of sustained pre-renal AKI, resulting in a deficient supply of oxygen and glucose to the tubular compartment. While in the first case, patient handling should address the cause of the primary tubular damage and its progression, in the second, management should aim at restoring renal blood flow and hemodynamics. Yet, distinction between cases through biochemical ratios and injury biomarkers may be difficult. Injury biomarkers long believed to be produced by damaged tubules and shed directly to the tubular lumen, including NGAL, TIMP-2 and IGFBP7, have been shown to reach the urine, instead, due to impaired tubular reabsorption [48,49,50]. Their renal excretion is, thus, not reflective of whether impaired reabsorption results from damaged tubules or from sublethal incompetence (or a combination of both), nor of whether tubular damage or dysfunction is a primary event or secondary to hypoperfusion. Accordingly, these classification criteria are limited to providing information on whether there is parenchymal involvement (i.e., mainly tubular damage or dysfunction) in the pathological process, regardless of its primary etiology.

Overall, our results provide a primary proof of concept for a new, potential AKI diagnostic strategy for the identification of the underlying pathological pattern, which is based on the combination of objective biochemical parameters rather than solely on anamnestic evaluation. The combination of several biochemical indexes may reduce or minimize the effects of confounding factors, and incorporation of urinary injury biomarkers may provide additional accuracy. However, our results are limited by the modest size of the study population. Accordingly, larger studies are necessary to confirm the present findings, as well as to identify the most suitable biochemical ratios and urinary injury biomarkers providing the highest diagnostic congruency and the strongest mutual reinforcement.

## 4. Materials and Methods

### 4.1. Patients and Protocols

A total of 53 volunteers suffering from AKI who were referred to the Nephrology Department (Salamanca University Hospital, Salamanca, Spain) through inter-Service consultation, and who provided written consent, were included in this study. All protocols were approved by the local Ethics Committee and were conducted according to the principles established in the Declaration of Helsinki (World Medical Assembly), the Council of Europe Convention on Human Rights and Biomedicine and the UNESCO Universal Declaration on the Human Genome and Human Rights; the requirements established in the Spanish legislation in the field of biomedical research, personal data protection and bioethics; as well as the provisions of the Law 14/2007 of 3 July, of Biomedical Research and RD 53/2013 of 1 February. Renal function was monitored by means of Cr_p_, and AKI was defined and classified according to the Kidney Disease: Improving Global Outcomes (KDIGO) criteria [51] from Cr_p_ and urine output data. Urine was collected upon admission to the Nephrology Department and was used to measure six renal injury biomarkers (as described below), namely N-acetylglucosaminidase (NAG), NGAL, KIM-1 [52,53], chaperonin containing TCP-1, subunit *eta* (TCP1-*eta*) [38,54], ganglioside activator protein 2 (GM2AP) [54,55,56] and transferrin [55,57,58,59].

Patients were classified as suffering from pre-renal or renal (i.e., intrinsic) AKI based on anamnestic and biochemical criteria. Each patient was classified independently with both criteria:

- The anamnestic criterion classified patients under pre-renal AKI when a decrease in circulating volume was suspected, (i) as per fluid loss following hemorrhage, diarrhea, vomiting, abundant debit by nasogastric tube, diuretics, osmotic diuresis, diabetes insipidus, adrenal insufficiency, fever, burns, tachypnea, etc.; (ii) due to extracellular fluid redistribution, as in edematous states, pancreatitis, peritonitis, intestinal obstruction, crush syndrome, etc.; or (iii) when symptoms of renal hypoperfusion were evident, as in patients with heart failure or shock, suspicion of renal vasoconstriction (as in hepatorenal syndrome, sepsis, use of alpha-adrenergic therapy or hypercalcemia) or drugs altering renal autoregulation (e.g., NSAIDs, calcineurin inhibitors, ACE inhibitors, ARA II, etc.). In these situations, arterial hypotension, orthostatism, and tachycardia may be observed. On examination, mucosal dryness, ocular hypotonicity, decreased central venous pressure or pulmonary capillary pressure, diuretic response to volume expansion and improvement after cause withdrawal also supported pre-renal classification. Renal hypoperfusion, mainly in severe or prolonged forms of ischemia, can condition ATN. Patients with hypotension during surgery, bleeding or sepsis have an increased risk of developing ischemic ATN, especially in the presence of other associated pathologies, such as previous chronic renal failure, diabetes mellitus, arteriosclerosis or malnutrition. Prerenal forms of AKI due to hypovolemia or decreased effective circulating volume due to heart failure or liver disease may also be perpetuated and lead to ischemic ATN. Clinically, it differs from prerenal ARF in that renal hypoperfusion causes damage to the tubular cells, and in that after establishing the appropriate treatment, there is no increase in diuresis nor a decrease in azotemia.
- The biochemical criterion was based on the following ratios: (i) Urinary creatinine/plasma creatinine ratio (Cr_u_/Cr_p_), with values > 20 indicating pre-renal AKI and <20 renal AKI. (ii) Fractional excretion of sodium [FENa = (Na_u_ × Cr_p_)/(Na_p_ × Cr_u_) × 100], with values < 1 indicating pre-renal AKI and >1 renal AKI. (iii) Renal Failure Index (RFI) = (Na_u_ × Cr_p_)/Cr_u_. with values < 1 indicating pre-renal AKI and >1 renal AKI [26,60,61,62,63]. Na_p_ and Na_u_ stand for plasma and urinary Na concentration, respectively, and Cr_p_ and Cr_u_ for plasma and urinary creatinine concentration. For the biochemical criterion, patients were classified as pre-renal or renal AKI when meeting at least two (of the three) ratios for pre-renal or renal AKI. Renal function and diagnostic data, as well as Na_p_, Na_u_ and Cr_p_, were obtained from the patients’ medical records. Cr_u_ was measured with a Quantichrom Creatinine Assay Kit (BioAssay Systems, Hayward, CA, USA) according to the manufacturer’s instructions.

### 4.2. Biomarker Measurement

NAG was quantified using a commercial N-Acetyl-β-D-glucosaminidase Assay Kit, (Diazyme, Poway, CA, USA) according to the manufacturer’s instructions. NGAL, KIM-1 and transferrin were measured with the following commercial ELISAs: Human NGAL ELISA Kit 036CE (BioPorto Diagnostics, Hellerup, Denmark), KIM-1 (human) ELISA kit ADI-900-226 (Enzo Life Sciences, Farmingdale, NY, USA) and Human Transferrin ELISA Quantitation Set E80-128 (Bethyl Laboratories, Montgomery, TX, USA), respectively. TCP1-*eta* and GM2AP were measured by Western blot. Briefly, 21 μL of urine from each patient was separated by acrylamide electrophoresis. Proteins were transferred to an Immobilon-P Transfer Membrane (Millipore, Madrid, Spain) and incubated with the following primary antibodies: (i) TCP1-*eta* antibody (Novus Biologicals, Littleton, CO, USA) and (ii) GM2AP (in-house polyclonal antibody, described in [56]). Membranes were then incubated with horseradish peroxidase-conjugated secondary antibodies, and chemiluminescent detection was performed with Chemidoc MP, (BioRad, Madrid, Spain). Bands were quantified with ImageLab software, (BioRad, Madrid, Spain) and normalized to the signal of three dilutions of positive control (as arbitrary units) conforming to a linear standard, all loaded in gels. The positive control consisted of a urine sample from a designated AKI patient with increased biomarker excretion, which was used as a trans normalization control in all experiments. In all cases, biomarker data values were normalized by their corresponding Cr_u_.

### 4.3. Data and Statistical Analysis

Frequencies and percentages for all of the categorical parameters were compared between the pre-renal and renal AKI groups, according to both biochemical and anamnestic classification criteria, using Pearson’s chi-squared or Fisher’s exact test. In the case of continuous variables, after verifying their non-normality using the Shapiro–Wilk test, they were compared using the Mann–Whitney U test. The diagnostic capacity of urinary biomarkers to differentiate patients with pre-renal AKI from those with renal AKI was evaluated using an ROC curve-based analysis [64]. Finally, all urinary biomarkers were included in a binary logistic regression analysis to build a mathematical model discriminating patients with pre-renal AKI from those with renal AKI.

The criterion for statistical significance was set at *p* < 0.05. All of the statistical analyses was performed with the IBM SPSS statistics software version 20 (International Business Machines, Armonk, NY, USA). IBM SPSS statistics software version 20, Microsoft Office Excel 2016 and PowerPoint 2016 (Microsoft, Redmond, WA, USA) were used to create the artwork and illustrations presented.

## Figures and Tables

**Table 1:** Patient characteristics per AKI type (i.e., pre-renal or renal) according to anamnestic and biochemical criteria. Data are presented as the median (minimum–maximum). ACEIs, angiotensin-converting enzyme inhibitors; AKI, acute kidney injury. ARBs, angiotensin II receptor blockers; NSAIDs, non-steroidal anti-inflammatory drugs.

Patient Characteristics	Biochemical Criterion			Anamnestic Criterion		
	Pre-Renal AKI (n = 25)	Renal AKI (n = 28)	*p*-Value	Pre-Renal AKI (n = 31)	Renal AKI (n = 22)	*p*-Value
Gender (male/female, %)	48.0/52.0	35.7/64.3	0.28	45.2/54.8	72.7/27.3	0.06
Age (years)	71.0 (27–92)	75.5 (40–89)	0.62	72.0 (27–92)	73.5 (40–89)	0.70
Obesity (no/yes, %)	66.7/33.3	94.1/5.9	0.13	75.0/25.0	88.2/11.8	0.62
Diabetes mellitus (no/yes, %)	72.0/28.0	60.7/39.3	0.56	67.7/32.3	63.6/36.4	0.78
Hypertension (no/yes, %)	12.0/88.0	25.0/75.0	0.30	12.9/87.1	27.3/72.7	0.29
Heart disease (no/yes, %)	52.0/48.0	67.9/32.1	0.27	58.1/41.9	63.6/36.4	0.78
Ischemic (no/yes, %)	60.0/40.0	67.9/32.1	0.58	64.5/35.5	63.6/36.4	1.00
Valvular (no/yes, %)	68.0/32.0	89.3/10.7	0.09	71.0/29.0	90.9/9.1	0.10
Smoking (no/yes, %)	78.3/21.7	76.0/24.0	1.00	81.5/18.5	71.4/28.6	0.50
Previous pharmacological treatment:						
ACEIs (no/yes, %)	64.0/36.0	71.4/28.6	0.77	61.3/38.7	77.3/22.7	0.25
ARBs (no/yes, %)	60.0/40.0	41.4/28.6	0.40	64.5/35.5	68.2/31.8	1.00
Diuretics (no/yes, %)	36.0/64.0	42.9/57.1	0.78	29.0/71.0	54.5/45.5	0.09
NSAIDs (no/yes, %)	81.8/18.2	73.1/26.9	0.51	76.9/23.1	77.3/22.7	1.00
Contrast medium (no/yes, %)	96.0/4.0	100.0/0.0	0.48	96.8/3.2	100.0/0.0	1.00
Plasma creatinine (mg/dL)	5.3 (1.7–12.5)	4.3 (1.9–13.5)	0.93	5.3 (1.7–13.5)	4.0 (1.9–9.5)	0.15

**Table 2:** Best logistic regression models for etiological (i.e., pre-renal and renal) AKI diagnosis based on urinary biomarkers. Cr_u_: urinary creatinine concentration. NAG: N-acetylglucosaminidase.

Biochemical Criterion				
Parameter	B	SD	Wald	*p*-value
Logistic regression analysis (only transferrin)				
Transferrin (ng/mg Cr_u_)	0.095	0.040	5.734	0.017
Constant	−1.209	0.540	5.009	0.025
Specificity: 81.8%; Sensitivity: 61.5%; Percentage of success: 70.8%				
Logistic regression analysis (transferrin and NAG)				
Transferrin (ng/mg Cr_u_)	0.095	0.040	5.510	0.019
NAG (IU/mg Cr_u_)	70.28	30.02	5.481	0.019
Constant	−2.376	0.809	8.622	0.003
Specificity: 77.3%; Sensitivity: 76.9%; Percentage of success: 77.1%				
**Anamnestic Criterion**				
No significant model was obtained for any of the biomarkers analyzed.				

**Table 3:** Contingency tables showing no statistically significant impact of the diuretic treatment on the etiological classification of AKI.

Presence of Diuretic Treatment		Number of Patients with Coincident Etiological AKI Classification (Pre-Renal/Renal)		*p*-Value
		Non-Coincident	Coincident	
**Any Diuretic**	No	2	19	0.46
	Yes	6	26	
**Thiazides**	No	5	26	1.00
	Yes	3	19	
**Loop diuretics**	No	4	35	0.19
	Yes	4	10	

## Data Availability

Data are available upon reasonable request.
